# Frequency of neck and shoulder pain and use of adjustable computer workstation among bankers

**DOI:** 10.12669/pjms.322.9067

**Published:** 2016

**Authors:** Maryam Shabbir, Sajid Rashid, Bilal Umar, Aqeel Ahmad, Sarah Ehsan

**Affiliations:** 1 Dr. Maryam Shabbir, PHD Scholar. Assistant Professor, Riphah College of Rehabilitation Sciences, Riphah International University, Lahore Campus, Pakistan; 2 Dr. Sajid Rashid, PhD Scholar, Principal, Multan College of Physiotherapy, Multan. Riphah College of Rehabilitation Sciences, Riphah International University, Lahore Campus, Pakistan; 3 Dr. Bilal Umar, PP-DPT, MS-)MPT. Senior Lecturer, Riphah College of Rehabilitation Sciences, Riphah International University, Lahore Campus, Pakistan; 4 Dr. Aqeel Ahmad, PhD Scholar. Assistant Professor, Isra University, Islamabad campus, Pakistan; 5 Dr. Sarah Ehsan, PP-DPT. Lecturer, Azra Naheed Medical College, Superior University, Lahore, Pakistan

**Keywords:** Computers, Musculoskeletal diseases, Shoulder Pain, Neck, Workplace

## Abstract

**Background & Objective:**

Neck and shoulder are the most susceptible areas for developing musculoskeletal symptoms among computer users. The modifiable risk factors for these work related musculoskeletal disorders include physical office environment and psychosocial work related factors. Computer workstation layout had been shown to be an important physical aspect of work environment that influences the upper quadrant symptoms. Our objective was to find the frequency of neck and shoulder pain and use of adjustable computer workstation among bankers of Islamabad/Rawalpindi/Multan

**Methods:**

A cross sectional study was conducted and 120 participants were questioned. Purposive sampling technique was used in this study. Maastricht Upper Extremity Questionnaire (MUEQ) was remodeled and important questions were extracted from its detailed version. The tool was then validated by taking expert opinion. Frequencies and percentages were calculated for categorical variables.

**Results:**

Pain in the neck during working hours was experienced by 71.67% of the respondents and 48.33% of the participants had experienced shoulder pain during working hours. Adjustable keyboards were used by 16.67% of respondents. Back care material was used by 40% bankers. Adjustable chairs were used by 95.83% of the participants. Only 3% of the bankers did not have chairs with adjustable heights. Chairs with adjustable armrests were used by 25% bankers.

**Conclusion:**

Neck and shoulder pain are common occurrences among bankers. Most of the components of workstations of bankers were adjustable but some of them still need attention.

## INTRODUCTION

Computers have become an integral part of offices and work places. However, there is also an increasing prevalence of upper quadrant symptoms with computer usage. Neck and shoulder are the most susceptible areas for developing musculoskeletal symptoms among computer users. The modifiable risk factors for these work related musculoskeletal disorders include physical office environment and psychosocial work related factors. Computer workstation layout had been shown to be an important physical aspect of work environment that influences the upper quadrant symptoms.^1^

Ergonomics is a relatively new concept in Pakistan yet to be considered an essential component of most enterprises.^2^ The nature of ergonomics performs a unique role in protecting human health and preventing health risks.^3^ Introduction of adjustable workstations is an important step in application of ergonomics.

A focus on following ergonomic principals can lead to a reduction in musculoskeletal problems and increase in productivity of the workers.^4^ The relevance of this science to design a workplace in terms of tasks of the employee, making use of tools and the environment is called ergonomic design. A good ergonomic design not only maximizes the capabilities of workers by increasing efficiency and job satisfaction, but also gives a payback to the company by decreasing the cost for health and absence of workers due to health conditions. In further terminology, ergonomics makes sure “appropriating the duty to the worker”.^2^

During the 21th century, the banking sector in Pakistan is witnessing a tremendous change because of globalization, liberalization and other worldwide proceedings. The banking sector in Pakistan has moved out through many reforms in view of the fact that nationalization banks took place in 1974. In 1991 momentous policy changes in the banking division were introduced in the structure of privatization, in order to offer enhanced services to consumers and to support a competitive environment. Globalization and privatization policy led the banking segment of Pakistan to provide the impetus for reform and adaptation to a competitive advantage in multinational companies as environment.^5^ It has been reported that there is an increased chance of developing upper limb symptoms due to the risk factors at workplace.^2^

The beginning of electronic banking reforms and widespread use of computers and setting up of ATMs nationally are changing model of bank employees working in Pakistan. Considerable changes have affected both directly and indirectly. These are common, monetary and psychosomatic subject of bank employees and Pakistan. These are the potential causes of occupational stress and related disorders among employees in the banking sector of Pakistan. Conventional wisdom suggests that due to internal and external changes, employees in the banking sector in Pakistan are facing high levels of stress. Extended Perceived stress in Pakistani bankers is causing them to suffer from exhaustion.^5^

A flexible work environment would have a positive influence on the productivity of the individuals. Neck and shoulder pain have been found to be associated with prolonged working hours and improper sitting postures.^4^ Sustained sitting postures and poor workstation designs have been found to be linked with development of musculoskeletal disorders among computer users. 4

Bankers have to work for prolonged durations on computer workstations and may have to work overtime as well. The knowledge of prevalence of neck and shoulder pain and the use of adjustable equipment will work as a basis for further studies on applying ergonomic interventions for bank environments. As such it is important to check the level of awareness of office/bank workers about ergonomics and to assess whether they are being provided with appropriate ergonomically designed equipment or not.

The objective of this study was to find the frequency of neck and shoulder pain and use of adjustable computer workstation among bankers of Islamabad/Rawalpindi/Multan

## METHODS

This observational cross sectional survey was conducted among 120 participants (bankers) from private and public sector banks of Islamabad/Rawalpindi/Multan in six months duration after the approval from ethical review committee of the respective settings. Purposive sampling technique was used. Participants were enrolled from National Bank, United Bank Limited, Habib Bank Limited, Faysal Bank, Bank Al Habib, Habib Metropolitan Bank, KASB Bank, Standard Chartered Bank, Dubai Islamic Bank, Summit Bank and Muslim Commercial Bank. Maastricht Upper Extremity Questionnaire (MUEQ) was remodelled and important questions were extracted from its detailed version.^6^ Validation of the modified form of questionnaire was done by expert opinion. The Cronbach’s alpha for the content validity was 0.73. Questionnaires were self-administered and in English language and were given to the bankers’ supervisors, Branch Managers after taking written permission from the Main Branches of above mentioned banks in North cluster; as well as verbal consent from the participants. All participants were Bank employees of 17^th^ grade or above, who worked on computers for at least 6 hours a day. Those who were doing Internship or not willing to participate were excluded from the study. Data was compiled and analysed using SPSS Version 17. Frequencies and percentages were used for categorical data.

## RESULTS

Out of 120 participants 48.33% were females. Mean ± SD for the age of participants for public and private sector bankers were 32.82±6.240 and 30.79±5.055 respectively. Out of 120 respondents 57 were from public sector banks and 63 were from private sector banks.

The highest level of education was Masters for 45.83% of the bankers. In response to ‘do you work over time’ 55% respondents responded affirmatively.

Adjustable keyboards were used by 16.67% of respondents. Back care material was used by 40% of bankers. Adjustable chairs were used by 95.83% of the participants. Only 3% of the bankers did not have chairs with adjustable heights. Chairs with adjustable armrests were used by 25% of the bankers.

Monitors with adjustable level were used by 72.2% respondents. The percentage of respondents who could move their elbows, wrists and knees freely was 65.83%, 68.33% and 73.33% respectively.

Pain in the neck during working hours was experienced by 71.67% of the respondents and 48.33% of the participants had experienced shoulder pain during working hours. During the non-working hours 78.34% participants responded a decrease in level of pain.

## DISCUSSION

The pre-requisite for the application of ergonomic design to workstations in offices/banks is to first evaluate and find out the present conditions and current use of ergonomically designed equipment and knowledge of ergonomics in office workers and bankers. This study was conducted to determine the level of awareness and to see whether ergonomically designed equipment is used by bankers or not. The results have shown that both private and public sector banks lag behind in terms of the knowledge and application of ergonomic design.

Office environment has been found to be an important factor in enhancing the productivity of office workers.^7^ A study conducted in Nigeria has shown that 3.4 percent of participants had the knowledge of ergonomics. The learning sector and medical professionals do not fair better as only 10 (1%) and 20 (2.1%), respectively were aware of ergonomics. Studies suggest that a low level of ergonomics awareness may be due to the verity that Nigerians were not familiar with the benefits derivable from ergonomics.^8^

Study conducted in banking institutions in Nairobi, Kenya indicated that most employees did not know about the application of ergonomics in banking institutions. There was a significant gender difference; Women (41.32%) were more aware of the applicability of ergonomic exercises as compared to men.^9^

The design of workstations in offices/banks can have a huge impact on health of the workers. Efforts are being made to create a working environment that would enhance the working capabilities of the workers and reduces the environment related risk factors for developing various musculoskeletal disorders. The application of ergonomic principles to the workstation design optimizes the relationship between the worker and his/her environment. Ergonomics is directly linked to the competence level of the employers.^10^

Our results are consistent with previous studies. A greater frequency of pain has been found to be associated with computer use for greater than five hours per day.^11^ Neck pain was prevalent among bankers of Dhaka and has been found to be associated with type of job, workstation design and job demand.^12^ Overall number of workshops on ergonomics was less for both sectors but private sector bankers had attended more workshops as compared to public sector bankers. So this emphasizes the need for conducting more workshops in order to raise awareness about the importance of ergonomics in health promotion and prevention of musculoskeletal disorders.

Another study conducted in Nairobi has shown that most of the computer using bank employees suffered from musculoskeletal problems.^13^ Strong association has been found between work related musculoskeletal disorders and improper workstation design in another study conducted on public and private sector bankers. 14

Less work has been done regarding the type of workstation designs used in banks in this setting. In order to ensure the nation wide application of ergonomics in banks it is important to conduct such studies on large scales.

## CONCLUSION

The study concludes that Neck and shoulder pain are common occurrences among bankers with a more frequent experience of neck pain during working hours as compared to shoulder pain. Most of the components of workstations of bankers were adjustable but some of them still need attention. As adjustability does not necessarily indicate a reduction in musculoskeletal pain, therefore there is a need for further in-depth ergonomic analysis and introduction of ergonomically approved workstations to the bankers in order to minimize the occurrence of neck and shoulder pain and improving their productivity at work.
